# Fundamental and Practical Feasibility of Electrocardiogram Reconstruction from Photoplethysmogram

**DOI:** 10.3390/s24072100

**Published:** 2024-03-25

**Authors:** Gašper Slapničar, Jie Su, Wenjin Wang

**Affiliations:** 1 Department of Intelligent Systems, Jožef Stefan Institute, Jamova cesta 39, 1000 Ljubljana, Slovenia; 2 Jožef Stefan International Postgraduate School, Jamova cesta 39, 1000 Ljubljana, Slovenia; 3 Department of Electrical Engineering, Eindhoven University of Technology, 5612 AZ Eindhoven, The Netherlands; j.su@student.tue.nl (J.S.); wangwj3@sustech.edu.cn (W.W.); 4 Biomedical Engineering Department, Southern University of Science and Technology, Shenzhen 518055, China

**Keywords:** photoplethysmography, electrocardiogram, discrete cosine transform, QRS reconstruction, feasibility, reliability, robustness

## Abstract

Electrocardiogram (ECG) reconstruction from contact photoplethysmogram (PPG) would be transformative for cardiac monitoring. We investigated the fundamental and practical feasibility of such reconstruction by first replicating pioneering work in the field, with the aim of assessing the methods and evaluation metrics used. We then expanded existing research by investigating different cycle segmentation methods and different evaluation scenarios to robustly verify both fundamental feasibility, as well as practical potential. We found that reconstruction using the discrete cosine transform (DCT) and a linear ridge regression model shows good results when PPG and ECG cycles are semantically aligned—the ECG R peak and PPG systolic peak are aligned—before training the model. Such reconstruction can be useful from a morphological perspective, but loses important physiological information (precise R peak location) due to cycle alignment. We also found better performance when personalization was used in training, while a general model in a leave-one-subject-out evaluation performed poorly, showing that a general mapping between PPG and ECG is difficult to derive. While such reconstruction is valuable, as the ECG contains more fine-grained information about the cardiac activity as well as offers a different modality (electrical signal) compared to the PPG (optical signal), our findings show that the usefulness of such reconstruction depends on the application, with a trade-off between morphological quality of QRS complexes and precise temporal placement of the R peak. Finally, we highlight future directions that may resolve existing problems and allow for reliable and robust cross-modal physiological monitoring using just PPG.

## 1. Introduction

The electrocardiogram (ECG) is considered as the gold standard in cardiac monitoring [1]. It can be used to verify a person’s heart rate (HR), heart rate variability (HRV), and together with a peripheral the photoplethysmogram (PPG) to compute pulse arrival time (PAT) [2]. The information it offers is much richer compared to an optical PPG. The ECG reflects details of a person’s systole and diastole in the form of the QRS complex, allowing for the monitoring of detailed cardiac activity and the detection of potential heart conditions or diseases, such as subtle arrythmias, tachycardia, bradycardia, tachyarrhythmia, hyperkalemia, and variations of cardiomyopathy [3]. The value of the ECG was recently confirmed for automated AI-based detection of at least 12 diseases, including some mentioned previously [4]. ECG monitoring is difficult in terms of devices and medical personnel effort. Subsequently, there are several challenges with ECG monitoring, which make it mostly a medical procedure that is less often conducted in an environment outside a hospital. Some challenges include the requirement of correct electrode placement and good skin contact using a gel, possible allergic reactions to gel, potential motion artifacts, and general cable clutter which limits the activity of the person [5]. Additionally, an ECG requires at least two leads to function, making it difficult to create a compact device unlike for the PPG, where a single small sensor can be embedded into a wearable [6].

The PPG, on the other hand, is a noninvasive measurement that also monitors cardiac activity, but via an optical approach, measuring the periodic changes in blood volume in the tissue, again corresponding to the cardiac activity of systole and diastole [7]. Although the PPG can be used to estimate HR, HRV [8], and even blood pressure (BP) from such blood volume changes [9], it does not capture the subtle detailed information related to each phase of the heart contraction present in the ECG, as the ECG contains more detailed information in the P-wave, the QRS complex, and the T-wave [10]. The ECG is fundamentally different from the PPG as it represents electrical rather than optical modality of the cardiac activity and thus carries largely different information useful for different purposes. However, considering the previously highlighted challenges of the ECG measurement, the PPG signal is relatively simpler to obtain, requiring only a light source and a photodiode, which can be implemented even in consumer wearables, without the need for expert placement. Additionally, it is more ubiquitous, as anyone with an optical sensor (e.g., a smartphone camera) can, in principle, capture PPG using this device. Subsequently, PPG allows for user-friendly long-term continuous cardiac monitoring, at the cost of additional detailed information contained in the ECG.

**Figure 1:**
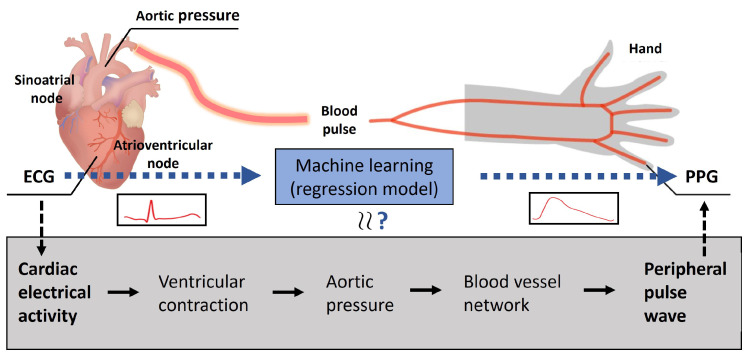
The relationship between the ECG and PPG based on the underlying cardiovascular system.

There exists a connection between the ECG and PPG, given that both monitor the same underlying cardiac activity, although via different modalities, as shown in Figure 1. Reconstruction of the ECG signal from PPG is thus attractive and would prove valuable, circumventing the challenges of ECG measurement while still allowing for additional cardiac activity detail to be derived simply from obtaining the PPG. Some early work in this area has already been conducted [11]; however, there are still questions that remain unanswered in terms of real-life feasibility, robustness, and reliability. For instance, does the shape of the reconstructed ECG signal maintain its physiological information and related medical value? Also, it is important to investigate whether the R peaks do not become too delayed to still be robust and reliable enough for applications wherein precise peak location is important (e.g., HRV monitoring, ratios between Q, R, and S locations, etc.). Importantly, the morphology of the waveform gives different information compared to just the location of the R peak, so we aimed to individually validate the R-peak location and waveform morphology in the ECG reconstruction. The relative cycle morphology can, for instance, be used to detect some cardiac diseases [3,4] (e.g., cardiomyopathy, ischemic heart disease, ventricular tachyarrhythmia, tachycardia, bradycardia, hyperkalemia, etc.), while the precise R peak location can be used for HRV or BP estimation via PAT, without the need for a perfect morphological reconstruction of the QRS complex.

While trustworthiness, reliability, and robustness are usually associated with explainable artificial intelligence (xAI) in terms of decision-making of the model [12], there is a need to consider them outside of black-box methods as well. Fundamental feasibility analysis and connection with underlying physiology—especially in light of the difficult reproducibility of the results plaguing digital medicine in recent years [13]—is vital and should not be overlooked. In this paper, we investigate and discuss both the fundamental and practical feasibility and trustworthiness of PPG-ECG reconstruction.

The rest of this paper is organized as follows. We start by investigating additional related work and identifying the important questions and challenges in such approaches in Section 2. We then describe a publicly available dataset [14], which we used for our evaluation, and proceed with the description of the PPG-ECG reconstruction methods we evaluated in Section 3 and Section 4. Finally, we describe the experimental setup and report the results in Section 5. We conclude with the findings, our contributions, and discussion about reliability and robustness of such reconstruction in Section 6 and Section 7, highlighting the important influence of cycle segmentation and alignment, as well as the evaluation scheme and metric used. We show that, while morphological properties of the QRS complex can be successfully modeled when PPG and ECG cycles are semantically aligned in regards to the cardiac systole, some important temporal physiological information is lost. Additionally, we show the importance of personalization for training a model to precisely reconstruct the waveform shape, hinting at the low possibility of training a truly robust and reliable general model.

## 2. Related Work

A pilot study on ECG reconstruction from PPG was conducted by Zhu et al. in 2019 [11]. This is the main starting point in this research domain and also the one that we used as the basis of our early work, to initially attempt to reproduce and then investigate and evaluate further. The authors proposed a linear transform using DCT coefficients of each PPG cycle to reconstruct the corresponding ECG cycle waveforms. The cycle pairs were semantically aligned to represent the same physiological phenomenon, meaning that the period between two systolic PPG peaks was aligned with the period between two ECG R peaks for training. This means that temporal information (e.g., interbeat intervals, peak locations, etc.) of the signal as a time series of cycles is lost. Their method was evaluated on a publicly available benchmark dataset in terms of relative root mean squared error (rRMSE) and Pearson’s correlation coefficient. All evaluation was intra-subject, where first 80% of the data was taken for learning and the last 20% for testing. They reported good results, achieving a lowest average rRMSE of 0.145 and correlation coefficient of 0.985.

There are, however, some concerns about the validation approach that was used and the heavy focus on morphology while neglecting the temporal properties, which we consider to be fundamental and critical for such reconstruction. When considering robustness, reliability and practical applications of such a reconstruction, which would be transformative for end-users, the following aspects should be reconsidered and investigated thoroughly:

- **Model personalization:** This is shortly discussed in the original paper; however, it is of vital importance when considering real-world application. The data of the same subject were used for training and testing, making each model completely personalized and requiring prior data of each given subject to train. In practice, such personalization would require a subject to calibrate the model with different HR data (e.g., at rest and immediately after activity).
- **Split of data:** Simply using the first 80% of the data for training does not give any information on the stability of the results, as this part of data can work very well or very poorly by chance, when perhaps the last 80% of the data would give notably different results. This also does not take into account temporal changes in cardiovascular dynamics (e.g., changes induced by HRV); in practice, it can be sometimes observed that a part of the data can consistently have the same HR, which only changes in some short sub-segments within a selected window. It would thus be better to consider a shorter window with overlapping, which can capture different parts of data for training and especially testing. The data could be sorted by HR and then the representation of different HRs in both the training and testing data can be assured.
- **Alignment of cycles:** When aligning individual cycles of ECG and PPG semantically, temporal information is lost and subsequently many potential use cases are no longer feasible (e.g., PAT, BP, HRV, etc.).
- **Evaluation metrics:** Using only correlation and rRMSE as the evaluation metrics exclusively focuses on the morphological quality of reconstruction, completely ignoring the temporal dynamics (e.g., delay between the ECG R peak and the PPG systolic peak). While the morphological quality can be useful on its own for detection of some diseases, the accuracy of reconstructing temporal properties should also be investigated as it is mandatory for monitoring of PAT, HRV, BP, etc.

The aforementioned pilot study spurred other researchers to investigate such reconstruction. Tian et al. [15] highlighted the limited representational power of the DCT used in the pilot study and proposed a novel approach using a cross-domain joint dictionary learning (XDJDL) framework. More precisely, they used k-SVD, which is a generalization of the k-means clustering algorithm to simultaneously optimize both the PPG and ECG representations and the corresponding transform between them. This allows for joint learning of a pair of signal dictionaries (ECG and PPG) and the transform between their sparse codes. The approach was validated on tens of thousands of cycle pairs from the MIMIC III database [16], covering a variety of cardiovascular conditions from many subjects. The evaluation schema of [15] were inter-subject or subject-independent, meaning that 80% of the whole data was taken for learning and 20% for testing. However, it is not completely clear how these data were sampled. Again, if it was taken randomly, this can be problematic for several reasons; first, it is rather volatile and a different split may produce rather different results. Second, if cycles are taken randomly, then very similar or almost identical neighboring cycles of the same subject can appear in both the train and test set, which means that overfitting is inevitable and the results are over-optimistic. They reported a lowest average rRMSE of 0.39 and a highest average correlation of 0.88, surpassing the inter-subject performance of the pilot study DCT approach.

Zhu et al. [17] have extended their work with analysis of reconstruction performance on different subject groups with different cardiovascular diseases in 2021. They found that the performance in terms of rRMSE and correlation coefficient is better for groups without cardiac diseases, hinting at the fact that, for cardiovascular conditions, it may be difficult to derive such a reconstruction.

Mehmood et al. [18] proposed using a smartphone camera as the sensor to record the fingertip and obtain rPPG, which is then fed to a custom convolutional neural network (CNN), which returns the SpO2, respiratory rate, and ECG of the subject. They built upon the DCT proposed in the pioneering work by adding a feed-forward neural network on top of it to facilitate learning in the ECG, thus replacing the ridge regression with a non-linear method.

Vo et al. [19] similarly used a black box end-to-end deep learning approach based on the Wasserstein generative adversarial network. They proposed this approach due to the lack of data and high data requirements of learning the transform function. They reported a Pearson’s correlation coefficient of 0.84 between the generated and ground-truth ECG waveforms, demonstrating the effectiveness of their approach.

In summary, related work in the area of ECG reconstruction from PPG is relatively limited, as it is a rather new and exciting area. Most work was conducted in the same research group; however, some fundamental questions remain unanswered, as highlighted earlier. These include the viability of a robust general model and oversimplified evaluation experiments (including split of data, potential overfitting, cycle alignment, and evaluation metrics focusing exclusively on morphology) that we investigated and answered in this paper. Some of the challenges were potentially resolved by black-box end-to-end deep learning approaches [19], but understanding the fundamental feasibility of such reconstruction and the physiological connection between PPG and ECG is pivotal before practical real-world applications.

## 3. Data and Denoising

**Figure 2:**
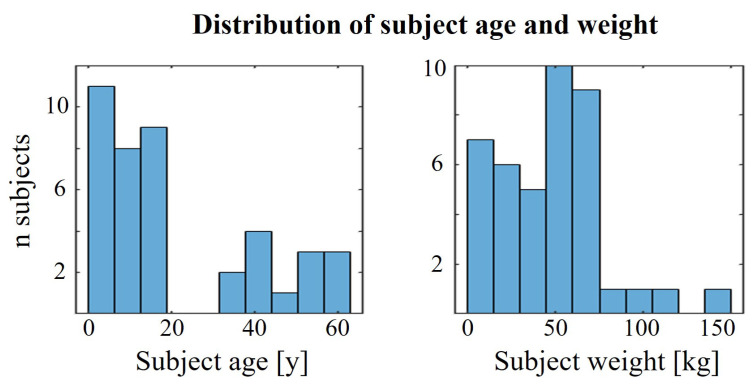
Histograms of patients ages and weights in the CapnoBase dataset used in our study [14].

The dataset used in our experiments is the publicly available CapnoBase TBME Respiration Rate Benchmark [14]. The same dataset was also used in the initial pilot study [11] and is thus suitable for a fundamental feasibility and comparative study. It contains 42 8-min synchronized PPG and ECG recordings recorded simultaneously during 29 pediatric surgeries and 13 adult surgeries containing reliable recordings of spontaneous or controlled breathing. The capnometric waveform was used as the reference gold standard recording for RR validation. Figure 2 shows the distribution of patients’ ages and weights, clearly showing both subject groups—pediatric and adult.

**Figure 3:**
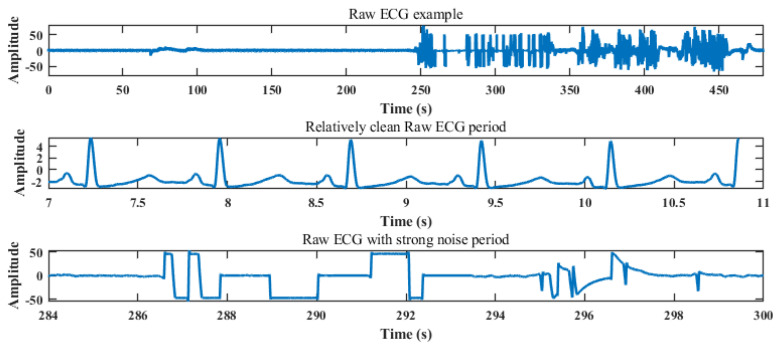
Raw ECG examples containing noise as well as stable periods, showing the apparent need for additional data cleaning.

As signals were recorded during surgery, they may contain irregular artefacts and noise. To obtain a relatively clean signal, denoising was first applied. Denoising consisted of two steps, namely manual distortion removal, and subsequent smoothing. All 42 subject data were initially verified manually. Figure 3 shows the raw ECG signal of one subject data as an example, highlighting the difference between the good and poor part of the signal. In this example, there are major distortions in the signal after the 250 s mark. Therefore, only the first 250 s were used for the following experiments, while the rest was manually removed. This same logic was applied to each of the recordings during manual inspection.

After this initial manual cleanup, a filtering step was then introduced to filter out small distortions due to high-frequency noise. The majority of ECG artifacts appear as a consequence of muscle activity, breathing, cable motion, etc. Traditionally, both high-pass and low-pass filters are used for ECG filtering [20]. As our aim was to smooth the high-frequency noise while introducing no phase delay due to the importance of a precise R peak location, we opted to use a Savitzky–Golay smoothing filter [21] with order = 3 and window length = 5. It is a digital filter that can be used to smooth the data without distorting the signal trend or introducing a phase delay. The overall shape and temporal properties of the signal are thus preserved, meaning the locations of the P wave, QRS complex, and T wave remain exactly the same, which is mandatory in light of keeping the important temporal physiological information intact.

## 4. Methodology

**Figure 4:**
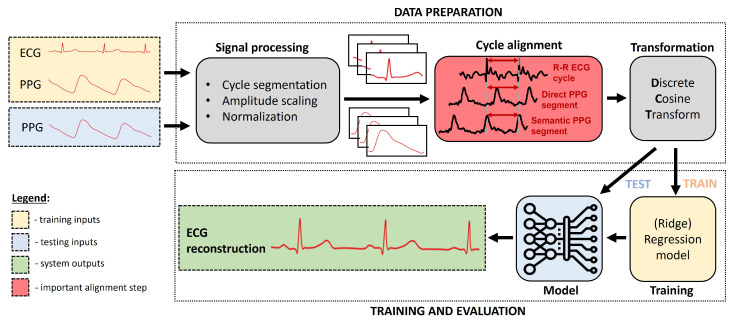
Architecture of the used pipeline, showing the inputs, outputs, and intermediate steps. The yellow blocks denote inputs used in training, the blue blocks denote the inputs used for testing, and the green block shows the system output. Other blocks are universal and always used.

The architecture employed in our research is based on fine-tuning of the system proposed by Zhu et al. [11] and comprises two main blocks, namely the signal pre-processing block and the model training block, as shown in Figure 4.

### 4.1. Signal Processing

In the following sections, we discuss the individual steps of our proposed and evaluated pipeline.

In the first part of the pipeline, the signal is pre-processed to obtain clean signals, which are then segmented into individual cycles.

#### 4.1.1. Cycle Segmentation

To learn the transfer function between PPG and ECG, the signals should be split into elementary units—cycles—that map one to another and correspond to the same cardiac contraction. The segmentation is based on the detection of reference points; for instance, systolic peaks in PPG. The signal between two systolic peaks corresponds to a single systole–systole cycle. We compare two segmentation options:

1. **Semantic segmentation/alignment [11]:** Originally, it was proposed that cycles are semantically aligned, meaning that synchronized PPG and ECG signals are segmented (e.g., by the systolic peaks) and then the delay information (pulse arrival time) is nullified by always taking the part of the ECG signal semantically corresponding to this same cycle, meaning between two R peaks.
2. **Direct segmentation/no alignment:** Since the temporal location of each cycle in relation to others is important and gives rich information about many physiological parameters (HRV, PAT, BP, etc.), we investigated the option of taking the part of the ECG signal that directly corresponds to the part of the PPG between two systolic peaks using the same timestamps. This way, the ECG reconstruction can retain the temporal information about R peak location.

The corresponding ECG cycle can either be the ECG signal in the same time period (between two PPG systolic peaks) or the ECG signal semantically corresponding to this same cycle, meaning between two R peaks. The choice of either using the corresponding temporal cycle or the corresponding semantic cycle in the ECG is important, as we show later in the results section.

#### 4.1.2. Temporal Scaling and Amplitude Normalization

The duration between two systolic peaks or two R peaks changes due to HRV, so the length of a cycle is not constant. To facilitate the transfer function learning process, we have to unify these durations numerically, while preserving the morphological properties. We used linear interpolation to equalize the length of all segmented cycles to *L* = 300 samples, which is a conservative value chosen based on an average cycle length of roughly 200 samples. The sampling frequency of the original data was 300 Hz. Finally, we also normalized the amplitude of all cycles.

### 4.2. Training a Reconstruction Model

To decrease the computational complexity and simplify the model, we used the discrete cosine transform (DCT) to obtain a compact representation of each cycle, both for PPG and ECG, respectively. DCT was shown to be useful for compressing ECG and PPG signals, as it allows for a compact representation while preserving important information [22]. We decided to use the amount of DCT coefficients that contained 99.98% of the energy contained in the signal. We decided to set a high threshold in order to keep as much morphological detail as possible, especially for the ECG. Empirical trial has shown that setting this threshold to 99% or less further decreases the required number of DCT coefficients at the cost of introducing a slight phase shift, which is undesired.

**Figure 5:**
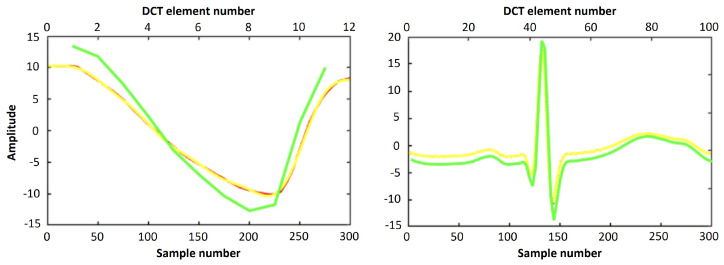
Original (pre-processed) signals in red, inverse DCT reconstruction with zero padding in yellow, inverse DCT reconstruction without zero padding in green.

Using the 99.98% threshold, we found that we required only 11 DCT coefficients to contain the PPG cycle information. The ECG, on the other hand, is morphologically more complex, so we required 100 DCT coefficients. Once the number of coefficients was determined empirically, we kept these values fixed throughout the experiments for all 42 subjects. Examples of the original pre-processed signals and reconstructions from their corresponding DCT representations using inverse DCT are shown in Figure 5.

Using the DCT transform representation, we trained a transfer function $f^{*}$ using ridge regression as defined in the original paper [11]:

(1) $$f^{*}=argmin(||X_{train}f-Y_{train}{\left|\right|}_{F}^{2}+{\gamma ||f||}_{F}^{2}),$$

where *x* is the PPG signal representation of dimensions $N_{x}\times L_{x}$, $N_{x}$ is the total number of cycles, $L_{x}$ is the length of the DCT representation for PPG determined previously, ${\left||input|\right|}_{F}$ is the Frobenius norm, and γ is a coplexity parameter controlling the shrinkage of *f*. *y* is the ECG signal representation of dimensions $N_{y}xL_{y}$, where $N_{y}=N_{x}$ (same number of cycles in the data) and $L_{y}$ is again the length of the DCT representation for ECG. The analytical solution to $f^{*}$ is then

(2) $$f^{*}={\left(X_{train}^{T}X_{train}+\gamma I\right)}^{-1}X_{train}^{T}Y_{train},$$

where *I* is the identity matrix. The ECG recontructions are then

(3) $$Y_{reconstructed}=X_{test}\cdot f^{*}.$$

Finally, the inverse transform iDCT is applied to the obtained coefficients, which are zero padded to the same length.

## 5. Evaluation and Results

### 5.1. Experimental Setup

We evaluated the reconstruction performance both intra-subject and inter-subject, to see whether a general model is feasible or whether reconstruction is person-specific. Two evaluation metrics were used, namely Pearson’s correlation coefficient between the reconstructed and ground truth ECG cycle (QRS complex), and the temporal delay between the reconstructed and real R peak in the QRS complex.

Two evaluation approaches were initially used. The first was five-fold cross-validation, which verified the stability of the results between different folds by sequentially taking different 80% of data for training and 20% for testing five times. We avoided overfitting and tested for robustness and generalization, as the data (signal cycles) were stacked in a way that the data of each subject were always kept sequentially together, without shuffling, meaning that when folds were split, the vast majority of the data in the train and test sets were from different subjects. A single subjects’ data do become split between the train and tests, but given the relatively large number of subjects this does not meaningfully skew the results. In Table 1, we refer to this as the general model.

Since the data were collected during operations in ICU, the HR of the subjects varied in bursts. For instance, most of the time, the HR was stable at 70 beats-per-minute (BPM), but a sudden change to 100 BPM for a short period occurred. As we want our model to successfully capture such changes (temporal “compression”, meaning systolic/R peaks were closer together), we designed a second evaluation scenario. The data were traversed with a sliding window of 20 cycles at a time, and each time the first 80% of the cycles was taken for training and the next 20% for testing. This was repeated until the end of the signal for each subject. Our aim was to ensure a roughly even representation of HRs between the train and test data, while trying to not overfit too much. We call this our 80–20 sub-sampling evaluation method and refer to it in Table 1 as the personalized model.

### 5.2. Evaluation Metrics

We used two evaluation metrics to measure the reconstruction performance in terms of morphology and preserved temporal properties of the important ECG reference point—R peak of the QRS complex. First is the Pearson’s correlation coefficient between the reconstructed and ground truth cycle, which described the similarity in terms of morphology and allowed for comparison with related work, which overwhelmingly uses it as the de facto metric. It is defined as follows:

(4) $$\rho \left(ECG_{R},ECG_{GT}\right)=\frac{cov(ECG_{R},ECG_{GT})}{\sigma \left(ECG_{R}\right)\sigma \left(ECG_{GT}\right)},$$

where ρ is the correlation coefficient, $ECG_{R}$ and $ECG_{GT}$ are the reconstructed and ground-truth cycle, cov(·) is the covariance, and σ(·) is the standard deviation, respectively.

Importantly, we added the second metric for evaluation of temporal position of the R peak in the reconstruction, where we simply computed the temporal difference (in seconds) between the R peak location in the reconstruction and ground truth as follows:

(5) $$R_{delay}=abs\left(R_{LOC}\left(ECG_{GT}\right)-R_{LOC}\left(ECG_{R}\right)\right),$$

where $R_{delay}$ is the temporal delay in seconds, $R_{LOC}$ are the relative locations of the R peak, abs(·) is the absolute value, and $ECG_{R}$ and $ECG_{GT}$ are again the reconstructed and ground-truth cycle.

We report the average Pearson’s correlation coefficient and R peak delay for each of the two evaluation scenarios and cycle segmentation methods described previously in Table 1.

**Table 1:** Results in terms of Pearson’s correlation coefficient and R peak delay using different cycle segmentation methods (direct vs. semantic) and evaluation experiments (general vs. personalized model).

	*Pearson’s Correlation Coeff.*		*R Peak Delay [s]*	
Segmentation	**General**	**Personalized**	**General**	**Personalized**
**Direct**	0.23	0.87	0.2	0.01
**Semantic**	0.86	0.99	0.40	0.40

### 5.3. Results

From Table 1, we can observe slightly better results using the personalized 80–20 subsampling method compared to five-fold CV. This is expected since the subsampling method is personalized while the five-fold CV is general. Additionally, we can observe a difference based on the ECG cycle segmentation method. When directly taking the ECG between two corresponding systolic PPG peaks, the correlation coefficient is lower while the R peak delay in the reconstruction is also low. On the other hand, using the semantic ECG cycle segmentation method, the correlation coefficient is very high, showing that the QRS complex is precisely reconstructed, but the R peak delay is also larger, meaning that the R location is shifted (centered), which is undesirable. This can be explained by the fact that, in semantic ECG cycle segmentation, we artificially align the peaks, which is not the case in the actual data. In the direct ECG cycle segmentation, we do not alter the QRS location, meaning we obtain a good temporal prediction of the R peak, but poorer QRS reconstruction since the QRS complex is located at slightly different location each time (due to HRV).

**Figure 6:**
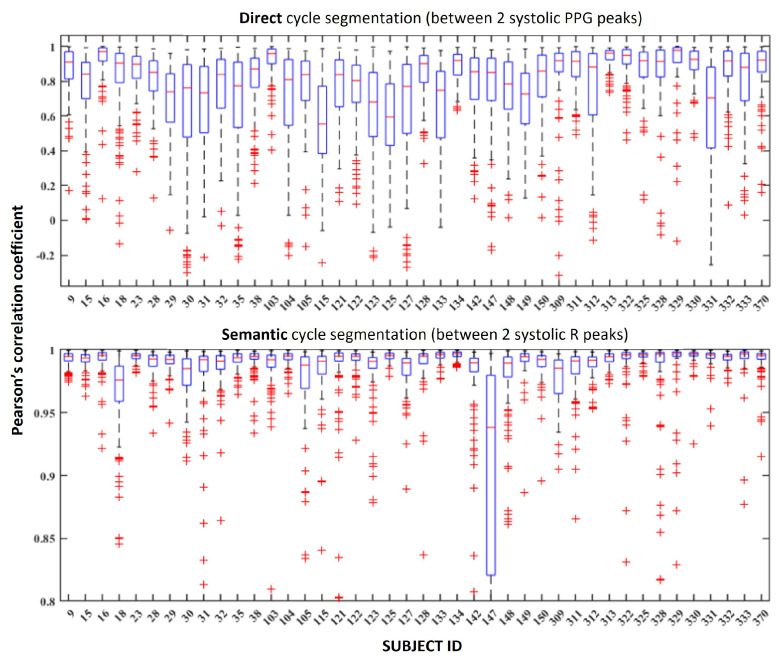
Per-subject boxplots of Pearson’s correlation coefficient using direct and semantic ECG cycle segmentation in the personalized evaluation experiment. Subject 147 was an outlier when doing semantic cycle segmentation, which can happen as a consequence of peak detector failing to correctly segment cycles in case of consistent severe distortions. This does not affect the direct PPG-based segmentation using different signal.

**Figure 7:**
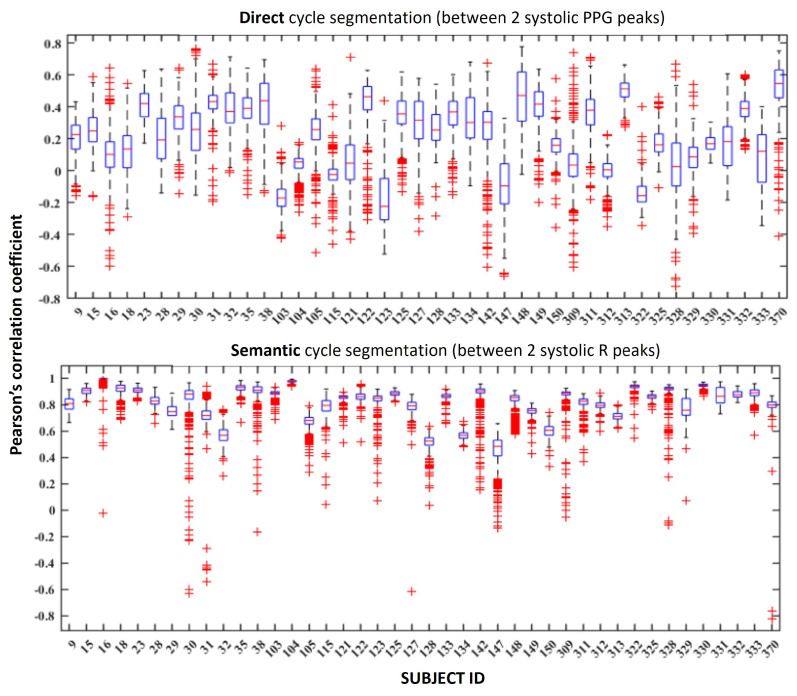
Per-subject boxplots of Pearson’s correlation coefficient using direct and semantic ECG cycle segmentation in the generalized evaluation experiment.

Using the personalized 80–20 subsampling evaluation method, we verified the correlation coefficient between reconstructed and ground truth ECG segment for each subject for each of the two cycle segmentation methods (direct and semantic). We see overall improvements across almost all subjects when using the semantic ECG cycle segmentation, as shown in Figure 6 and Figure 7.

**Figure 8:**
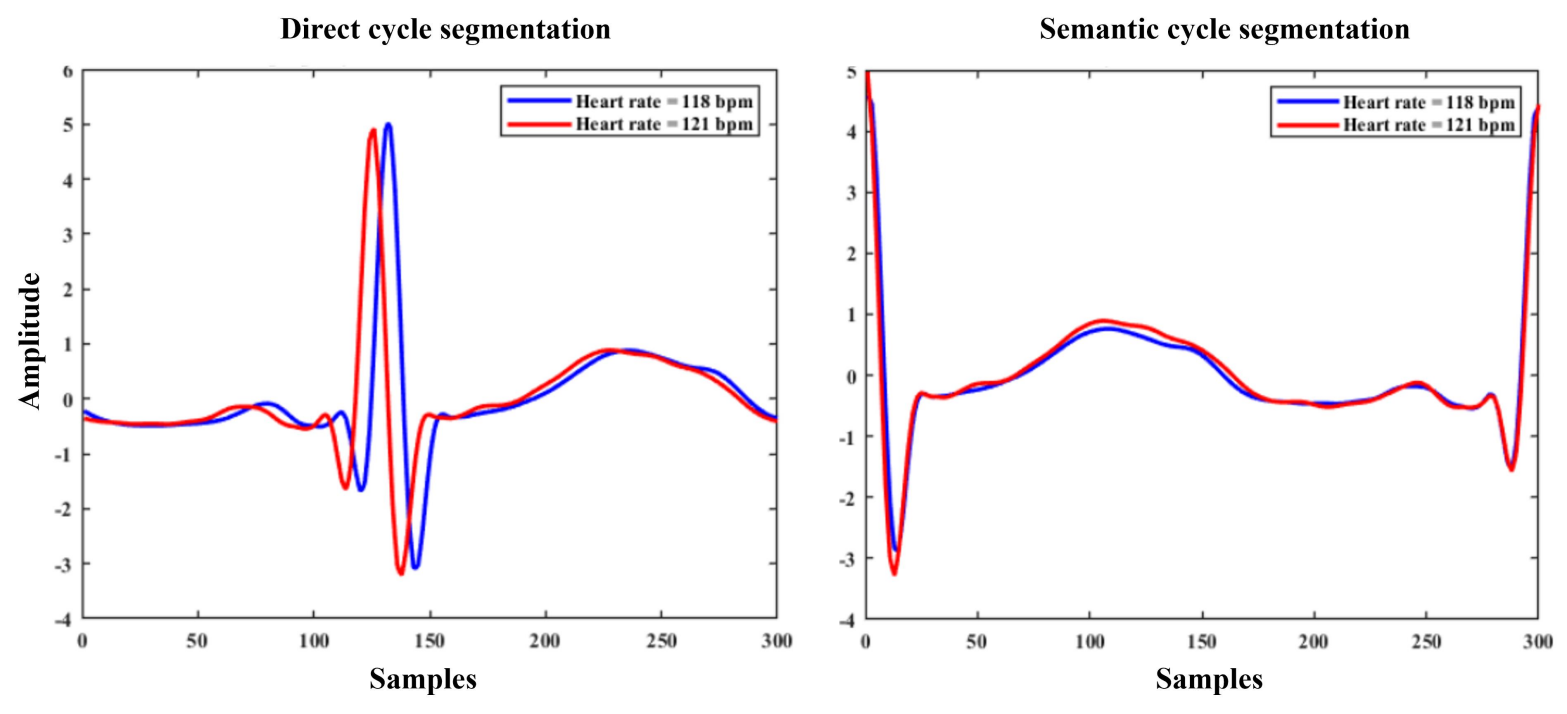
The plots compare different segmentation methods at different HRs. Left plot shows two ECG cycles of a subject at different HRs when using direct segmentation based on systolic peaks. The right plot shows the same cycles when using semantic (aligned) segmentation based on ECG R peaks. We can see the temporal shift between the two when using the first method, while the second preserves the shape to be almost the same.

We can observe consistent overall increases in correlation coefficients when using the semantic ECG cycle segmentation, even for subjects that perform worse in the direct segmentation (e.g., subjects 133 and 331). We investigated the subjects that performed worse in the direct cycle segmentation and found that these typically had larger variations in HR. This explains improved performance with semantic cycle segmentation, as we manually aligned the cycles and the model had an easier time creating a reconstruction. When we use direct segmentation, the PPG and ECG cycles are misaligned, which was difficult for the model to re-create, as shown in Figure 8.

**Figure 9:**
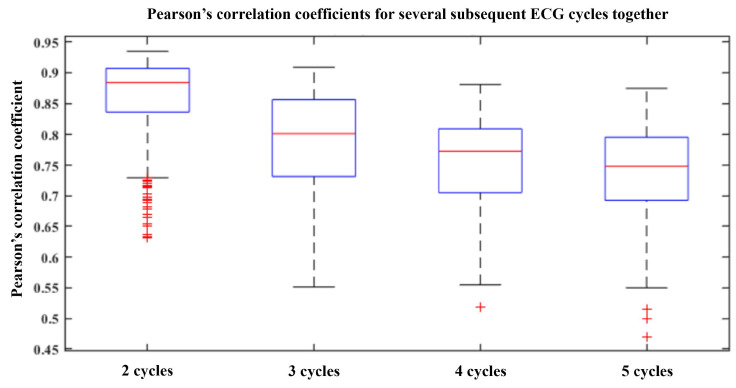
Boxplots showing decreasing Pearson’s correlation coefficients when we concatenate several subsequent cycle reconstructions. Due to accumulation of temporal misalignment, the value decreases the more cycles we add.

This indicates that the Pearson’s correlation coefficient may be an over-optimistic metric when using the semantic segmentation and manual alignment of cycles. Such a method is good when we want to evaluate the quality of the complex QRS reconstruction morphology individually, but when looking at several subsequent cycles together, we rapidly lose detailed peak positioning and important information about HRV or the ability to measure precise PAT for BP estimation. This was further confirmed by verifying the Pearson’s correlation coefficient when looking at reconstruction of longer periods of the ECG, containing several cycles. In Figure 9, we see that, the lower the correlation coefficient, the longer the reconstructed segment. More specifically, we reconstructed each cycle and then concatenated an increasing number of reconstructions and computed Pearson’s correlation coefficient on this longer segment. Since the cycles in the concatenation become increasingly temporally misaligned with the addition of each additional cycle, we obtain increasingly lower overall correlation despite relatively high correlation values for individual cycles.

We observed overall worse performance when trying to train a more generalized model, both in terms of correlation coefficient as well as R peak delay. There are several factors that should be considered when attempting to train a general model. We considered the age, weight, and HR of an individual. Using thorough empirical inspection, we found that differences in age and weight often cause some change in the morphology of the ECG, while different HRs (to otherwise similar subjects in terms of age and weight) cause a temporal shift of the QRS complex.

Considering these observations, we investigated the feasibility of training a robust general model via a five-fold CV (without shuffling), as well as using a leave-one-subject-out (LOSO) evaluation scheme. In the latter, we used the data of n−1 subjects for training and the remaining subject for testing, meaning no personalization was conducted. The boxplots showing Pearson’s correlation coefficients of left-out subject ECG cycle reconstructions with their reference ground truth are shown in Figure 7.

**Figure 10:**
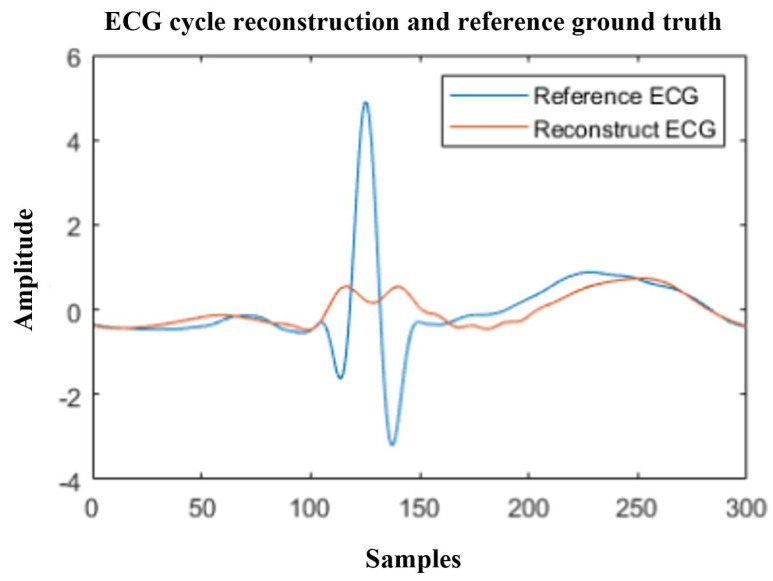
Example poor reconstruction (red) and reference ECG cycle (blue) in a LOSO experiment when using direct cycle segmentation.

The results of the five-fold CV for each of the two cycle segmentation methods are given in Table 1. Comparing the LOSO evaluation with the five-fold CV, we can once again observe that using the direct cycle segmentation method yields worse correlation coefficients compared to using semantic segmentation, as in the five-fold CV. Furthermore, we see that the performance when training a more general model is overall worse for both segmentation methods, compared to the personalized 80–20 subsampling technique shown in Figure 6. This degradation shows that reconstructing a perfectly correct morphological shape of the QRS complex is difficult for new unseen subjects, even when the cycles are semantically aligned. The result indicates that the precise details of the QRS complex are subject-specific to an extent and should be investigated further. Some correlations remain relatively high, so in some cases reconstruction is somewhat feasible, but overall the details of QRS morphology are lost. This is especially obvious when using direct cycle segmentation, as shown in an example in Figure 10.

As observed in Figure 10, the reconstructed waveform morphology can be rather poor in some cases when using direct cycle segmentation. While the P and S wave are relatively clear, the model only managed to reconstruct general trends in the area of the QRS complex, lacking morphological shape. This makes it unusable for the detection of conditions relating to QRS morphology, but we can observe some variation modeled in the QRS area, with good localization. This shows that, with direct cycle segmentation, informative temporal properties can be deduced, but such an approach should not be used for diagnoses relating to QRS morphology.

**Figure 11:**
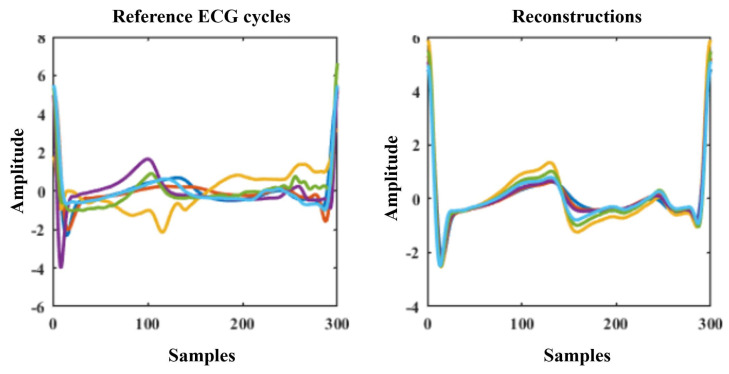
Example reference ECG cycles (**left**) and their corresponding reconstructions (**right**) in a LOSO experiment when using semantic cycle segmentation.

While correlation degradation in a LOSO experiment is severe for the direct cycle segmentation method, the semantic cycle segmentation maintains surprisingly high correlations. We thus compared the reconstructed and reference ECG signals of this segmentation method in more detail, by randomly selecting five subjects shown in Figure 11.

When comparing to direct cycle segmentation, we see that the morphology of the ECG cycle between two R peaks is reconstructed much better comparatively, although an overall similar general shape is always reconstructed, meaning that subject-specific details are once again lost. These reconstructions thus cannot and should not be used for precise morphological analysis or prediction of diseases where slight morphological changes are the main indicator.

## 6. Discussion and Limitations

Our aim was to investigate the feasibility, reliability, and robustness of ECG reconstruction from PPG using a pioneering approach reported by Zhu et al. [11]. We showed that, while morphological QRS reconstructions can be good when using manual semantic cycle alignment, there are several important things to consider when attempting to bridge the optical and electrical modality for cardiac activity before such a system can be considered reliable, robust, and trustworthy for practical applications.

First, since the method is cycle-based, it is very important how the cycles are segmented. When directly taking the ECG part between two systolic PPG peaks, the reconstruction performance is worse in terms of Pearson’s correlation coefficient; however, the temporal positioning of the systolic R peak in the ECG is better localized. This means that, when dealing with applications that require precise peak localization, such as HRV analysis or PAT computation for BP estimation, such a segmentation approach is better, despite lacking the QRS complex details in the reconstruction. On the other hand, using aligned cycles (between two systolic PPG peaks and between two ECG R peaks), the QRS complex reconstruction is better in terms of morphology, but loses important systole localization. Thus, there is an inherent trade-off between the cycle segmentation methods—direct segmentation better preserves temporal systole information while semantic segmentation with cycle alignment is better at reconstructing the QRS complex morphology.

Second, we found that a personalized approach using 20% of the sub-sampled data of the test subject in training achieves better performance in terms of correlation as well as R peak delay (depending on the cycle segmentation method), when personalization is performed in a way so as to capture different HRs in the training data as well. On the other hand, a general model performs worse, both in a five-fold CV (without shuffling) as well as a LOSO experiment. This reinforces the existing results from related work [17,23], showing that cardiovascular dynamics are person-specific and it is not feasible to expect superb performance for a new subject using a completely general model.

We replicated and extended existing pioneering methods, as our aim was to better understand fundamental feasibility, reliability, and robustness of such cross-modality reconstruction first. Thus, future work could offer improvements in terms of models and data used, especially given the fact that the currently proposed models are only linear, while the relationship between PPG and ECG is complex. This was partly addressed in recent work by other authors who used non-linear models [24]; however, the black-box nature and high overfitting capabilities of neural networks again circumvent the required fundamental understanding of feasibility of such cross-modality reconstruction, while adding an additional layer of uncertainty in terms of inference explanation. While such models can perform well and surpass traditional approaches in terms of raw numerical performance, they should consistently be accompanied with explainability analysis and mechanisms that could allow for healthcare professionals to be included in the loop.

Our position is that initial early work offers great promise and could importantly contribute to advances in healthcare monitoring, as obtaining two physiological modalities from a single sensor would be invaluable, as it would improve both subject comfort and adherence toward measurement, while offering richer information. However, we believe that it is crucial for future work in this cross-modal reconstruction to consider and investigate the discovered and reported challenges and pitfalls, as their resolution is mandatory before feasible practical implementation.

## 7. Conclusions

We investigated the fundamental and practical feasibility of cross-modal ECG reconstruction from PGG by extending and robustly evaluating an early proposed approach [11]. We found important facts and limitations that must be considered and resolved before robust and reliable practical applications, such as the difficulty of training a general reconstruction model due to subject-specific hemodynamics. Furthermore, we showed that, while the QRS morphology reconstruction can be good with certain specific pre-processing methods, the temporal properties of the ECG as a time series become lost in the reconstruction process, which further limits the practical applications (e.g., HRV, PAT, PTT, etc.) and trustworthiness of such a system.

The contribution and significance of this work lies in highlighting and empirically demonstrating limitations of current state-of-the-art approaches, focusing on unresolved challenges in terms of data segmentation, simplicity of linear models, and robustness of evaluation experiments, both intra and inter-subject. While such challenges can be potentially resolved by black-box approaches, they should be explicitly accounted for and understood, before such transformative monitoring technology can be put to practical and clinical use.

Despite existing limitations, there is great potential and significance in such cross-modal reconstruction for an easier and faster detection of diseases that manifest in QRS morphology anomalies, such as cardiomyopathy. Initial prototype approaches [11] used a linear model and specific data pre-processing that allowed for good reconstruction in specific conditions. Recent state-of-the-art approaches [18] built upon early approaches by adding non-linear black-box ANN models, which have greater modeling power and appear better-suited for ECG reconstruction from PPG. While such models provide impressive performance both in estimation of physiological parameters as well as ECG reconstruction, surpassing the early approaches, they should be extended with explainability and interpretability mechanisms that show or enable explicit understanding of the reconstruction.

Moreover, ANNs are infamous for their overfitting capabilities, which includes overfitting to a specific dataset, so evaluation should be expanded from proprietary datasets [18] to publicly available ones, preferably with a broad spectrum of people with different physiological states, such as the databases available in Physionet [25]. Since physiological state varies in time, especially long-term with aging, it would make sense to investigate the possibility of training models specific to age group, or add age and potentially other demographic features explicitly as inputs. It is plausible to assume such more specialized models could offer better reconstructions for specific groups of subjects.

Additionally, ECG reconstruction from longer segments should also be further investigated to potentially successfully model inter-beat interval temporal properties and extend practical application possibilities via applications like stress monitoring using HRV [26].

## Data Availability

Data are contained within the article.
